# Mapping the Knowledge Structure of Image Recognition in Cultural Heritage: A Scientometric Analysis Using CiteSpace, VOSviewer, and Bibliometrix

**DOI:** 10.3390/jimaging10110272

**Published:** 2024-10-26

**Authors:** Fei Ju

**Affiliations:** College of Art & Design, Nanjing Forestry University, Nanjing 210037, China; jufei1986@njfu.edu.cn

**Keywords:** cultural heritage, image recognition, deep learning, artificial intelligence

## Abstract

The application of image recognition techniques in the realm of cultural heritage represents a significant advancement in preservation and analysis. However, existing scholarship on this topic has largely concentrated on specific methodologies and narrow categories, leaving a notable gap in broader understanding. This study aims to address this deficiency through a thorough bibliometric analysis of the Web of Science (WoS) literature from 1995 to 2024, integrating both qualitative and quantitative approaches to elucidate the macro-level evolution of the field. Our analysis reveals that the integration of artificial intelligence, particularly deep learning, has significantly enhanced digital documentation, artifact identification, and overall cultural heritage management. Looking forward, it is imperative that research endeavors expand the application of these techniques into multidisciplinary domains, including ecological monitoring and social policy. Additionally, this paper examines non-invasive identification methods for material classification and damage detection, highlighting the role of advanced modeling in optimizing the management of heritage sites. The emergence of keywords such as ‘ecosystem services’, ‘models’, and ‘energy’ in the recent literature underscores a shift toward sustainable practices in cultural heritage conservation. This trend reflects a growing recognition of the interconnectedness between heritage preservation and environmental sciences. The heightened awareness of environmental crises has, in turn, spurred the development of image recognition technologies tailored for cultural heritage applications. Prospective research in this field is anticipated to witness rapid advancements, particularly in real-time monitoring and community engagement, leading to the creation of more holistic tools for heritage conservation.

## 1. Introduction

Cultural heritage is a valuable asset that represents the historical, cultural, and social values of civilizations. The preservation and study of cultural heritage have gained increasing attention globally, driven by the need to safeguard these assets from natural and human-induced threats, such as environmental degradation, urban development, and armed conflicts [1,2,3]. In this context, the application of advanced technologies, particularly image recognition, has emerged as a transformative approach in the field of cultural heritage preservation and analysis [4]. Image recognition, a subset of computer vision, involves the identification and classification of objects, features, and patterns within digital images, enabling the detailed documentation, monitoring, and analysis of cultural heritage artifacts and sites [5].

Among the various methods in image recognition, deep learning has proven to be one of the most effective approaches due to its ability to automatically extract features from complex visual data, making it particularly well-suited for heritage-related applications [6]. Deep learning, particularly Convolutional Neural Networks(CNN) [7,8] algorithms, excels at processing complex visual data and extracting relevant features automatically, which has made it indispensable for cultural heritage tasks such as artifact recognition [9,10], heritage restoration [11], and multimedia cultural data classification [12,13]. By enabling precise detection and analysis of heritage artifacts [14], image recognition serves as a foundational technology for digital preservation strategies, ensuring that detailed records of cultural sites and objects can be maintained and protected for future generations [15]. Over the past few decades, the integration of image recognition techniques has revolutionized how researchers and conservators approach cultural heritage studies, from automating the identification of architectural elements to reconstructing ancient artifacts and even exploring the Potential of Visually-Rich Animated Digital Storytelling for Cultural Heritage [16,17].

Image recognition and classification in the cultural heritage domain is a complex task, given the varied nature and conditions of artifacts and sites [18,19]. Recent advancements have demonstrated that for image classification tasks involving small cultural heritage datasets, Random Forest and Multilayer Perceptron classifiers are particularly suitable due to their robustness and flexibility in handling diverse features [19]. Furthermore, image recognition techniques based on Convolutional Neural Networks (CNNs) have been applied to the digital preservation of intangible cultural heritage (ICH) crafts, creating specialized communication platforms that enhance community cohesion by bringing together users from diverse backgrounds [20]. Additionally, a CNN-based image recognition model, integrated with wireless networks, has been employed to effectively describe and extract visual features of images, such as those of ethnic costumes, enabling efficient retrieval and fostering a better understanding of cultural diversity [21]. Moreover, image recognition methods have been adapted to specific conservation challenges, such as identifying biological pollutants that threaten heritage sites. For instance, a pre-trained classifier utilizing Support Vector Machines (SVMs) with a Radial Basis Function (RBF) kernel has been successfully used to classify types of biopollutants on cultural heritage monument images, providing a system for the early detection and management of such threats [22].

Despite the significant progress in this field, several challenges remain, including the requirement for interdisciplinary collaboration to effectively apply these technologies. Furthermore, the diversity of research output, methods, and applications in this area necessitates a comprehensive bibliometric analysis to understand the current state of research, identify key contributors, and map the evolving trends and hotspots.

In recent years, the literature analysis in the field of cultural heritage research has been divided into two main categories: systematic reviews and bibliometric analyses. Systematic reviews focus on research methods such as visualization, digital interaction, digital museums, remote sensing, and material science in the context of cultural heritage. Vlachos, Apostolos reviews recent Augmented Reality applications, emphasizing the use of camera-based tracking and handheld devices [23]. Okanovic, Vensada et al. discuss digital storytelling techniques that enhance user engagement through interactive multimedia [24]. Crane, Gregory et al. highlight the challenges in developing cultural heritage digital libraries, stressing the need for tailored collections and audience engagement [25]. Chen, Fulong et al. explore the advantages of spaceborne synthetic aperture radar (SAR) for subsurface imaging and landscape archaeology in heritage sites [26]. Casadio, Francesca et al. review the growth of Raman spectroscopy in art and archaeology, including its advanced uses in material identification and analysis [27].

Bibliometric analyses, on the other hand, focus on trends and research hotspots such as image analysis, artificial intelligence, visualization techniques, GIS applications, tourism, and cultural sustainability. Zhao, Yan et al. analyze Thangka image research, summarizing development trends and key contributors [28]. Dang, Xinyuan et al. provide a detailed overview of digital twin applications in Chinese heritage sites [29]. Boboc, Razvan Gabriel et al. review augmented reality applications in cultural heritage, identifying trends like 3D reconstruction and digital heritage [30]. Zhang, Jingru et al. analyze VR, AR, and MR technologies in cultural heritage to guide future research and practice [31]. Tang, Yuchen identifies 3D modeling and digital management as key topics in cultural heritage visualization research [32]. Huang, Yong highlights the importance of data integration and interdisciplinary collaboration in GIS applications in heritage studies [33]. Zhang, Sunbowen et al. examine cultural heritage tourism research, identifying key influences and future directions [34]. Aboulnaga, Mohsen et al. investigate how advanced technologies support cultural heritage conservation and climate resilience according to UNESCO’s Thematic Indicators [35].

These studies provide valuable insights into the evolving landscape of cultural heritage research. However, in the field of image recognition for cultural heritage, bibliometric analyses are still lacking, with only a few systematic reviews available, primarily focused on image classification and 3D object retrieval. Bellavia, F. offers a detailed overview of image-matching techniques within cultural heritage contexts [36]. However, their analysis reveals persistent challenges, such as the inability of current algorithms to handle image-matching tasks under difficult conditions like occlusions, lighting variations, and surface degradations, which are often encountered in real-world heritage sites. Bengamra, Siwar et al. present a comprehensive review of object detection methods for visual art and acknowledge that the diversity of artistic styles, along with the lack of sufficient annotated datasets, limits the performance of deep learning methods [37]. These limitations hinder the adaptability and generalizability of detection systems across various forms of cultural heritage. Savelonas, Michalis A. et al. focus on 3D object retrieval based on partial queries, highlighting significant advancements in retrieval methods [38]. However, they point out that existing techniques struggle with scalability and accuracy when dealing with fragmented or incomplete cultural heritage objects, which is a common challenge in archaeological data.

Despite the growing body of research on image recognition in cultural heritage, there is still a notable gap in the interdisciplinary integration of emerging AI technologies, environmental monitoring, and social policy-making. Existing studies often focus on isolated technological applications or specific fields without a comprehensive analysis of how these areas intersect. This study aims to bridge this gap by employing advanced bibliometric techniques to map the knowledge structure in cultural heritage image recognition. By synthesizing insights from multiple disciplines, this research provides a comprehensive analysis of how AI, deep learning, and other emerging technologies intersect with broader environmental and social factors, offering new perspectives on the evolving role of technology in cultural heritage preservation and analysis.

This study aims to fill this gap by conducting a systematic bibliometric analysis of publications on image recognition in cultural heritage from 1995 to 2024, using tools such as CiteSpace (version 6.3.R3), Bibliometrix (R package version 3.1.5), and VOSviewer (version 1.6.20). The primary objectives of this study are threefold: (1) to quantitatively and qualitatively analyze the temporal characteristics of publications in the field of image recognition for cultural heritage; (2) to examine the current status of author collaboration, institutional affiliations, national contributions, and cooperation networks within the literature; and (3) to identify research hotspots and predict future development trajectories in this area. The findings of this study aim to provide an objective and comprehensive understanding of the research landscape and future directions in the field of image recognition for cultural heritage preservation. This, in turn, will assist scholars and policymakers in gaining insights into the current state and depth of research in this domain. Additionally, the study’s results could offer valuable references for future research and policy formulation, thereby promoting the continued advancement of this field.

## 2. Materials and Methods

### 2.1. Data Source

The data used for analysis were obtained from the Web of Science Core Collection (WoSCC) database, comprising 586 articles retrieved on 15 August 2024. The WoSCC database is recognized as a reliable source for accurate and complete citation information [39]. The search string was (TS = (“heritage” OR “cultural heritage”) And TS = (“image recognition” OR “recognition”). The time span of the data was from 1 January 1995 to 15 August 2024, with “Article” or “Review” document types selected. Repetitive and irrelevant articles were filtered out. In the end, a total of 574 pertinent original articles were retained and exported in plain text files in the “Full Record and Cited References” format. Data cleaning involved removing duplicate entries, correcting inconsistencies in author names and affiliations, and standardizing terms to ensure data quality and reliability.

### 2.2. Data Analysis Methods

Bibliometric analysis involves both qualitative and quantitative assessments of the published literature. By extracting valuable visual insights from extensive bodies of the literature, bibliometric analysis enhances the rigor of research, making it a vital tool for scholars and policymakers [40,41]. This approach offers objective references for bridging the gap between theory and practice by summarizing empirical evidence from different disciplinary perspectives. This approach offers objective references for bridging the gap between theory and practice, particularly in the underexplored area of interdisciplinary research in cultural heritage image recognition. Previous studies have predominantly focused on isolated technologies or fields, lacking a comprehensive view of the interconnections between AI technologies, environmental monitoring, and social policies. By using bibliometric analysis to systematically map these evolving relationships, our study addresses this critical gap, offering a novel holistic understanding of how interdisciplinary efforts shape the future of cultural heritage preservation.

Furthermore, it provides new perspectives on the knowledge structure and development of scientific fields through data mining, information processing, and visualization techniques [42]. Scientific knowledge mapping, one of the commonly used techniques in bibliometric analysis, uses network visualization software to analyze the social and structural relationships among different research components, such as authors, institutions, countries, journals, keywords, and themes [43,44,45]. This method has been widely applied in various cultural heritage research areas, including immersive technologies, AVS (active vision system) [46], education [47], GIS [33], visualization [32], urban renewal [48], tourism [34], and architecture [49].

The existing mature bibliometric tools include CiteSpace, VOSviewer, Bibliometrix, HistCite, SciMAT, Ucinet, and SCI2. Among these, this study selected CiteSpace, Bibliometrix, and VOSviewer due to their complementary features and strengths, which enable a comprehensive mapping of the knowledge structure of cultural heritage image recognition and elucidate the interrelations among research outcomes over the past 29 years (1995–2024). The bibliometric analysis was conducted in three main stages: data preprocessing, data analysis, and visualization of results.

(1) CiteSpace was utilized to detect research fronts and emerging trends through co-citation analysis, keyword co-occurrence networks, and citation burst detection [50]. This software helps identify pivotal studies, authors, and evolving themes in the field;
(2) Bibliometrix, an R package, provided advanced bibliometric and network analysis capabilities, including the examination of collaborative networks among authors, institutions, and countries, as well as thematic mapping and trend analysis [51];
(3) VOSviewer was employed to visualize bibliometric networks, including keyword co-occurrence, co-authorship, and co-citation networks, using clustering techniques to uncover research hotspots and the intellectual structure of the field [52].

**Figure 1:**
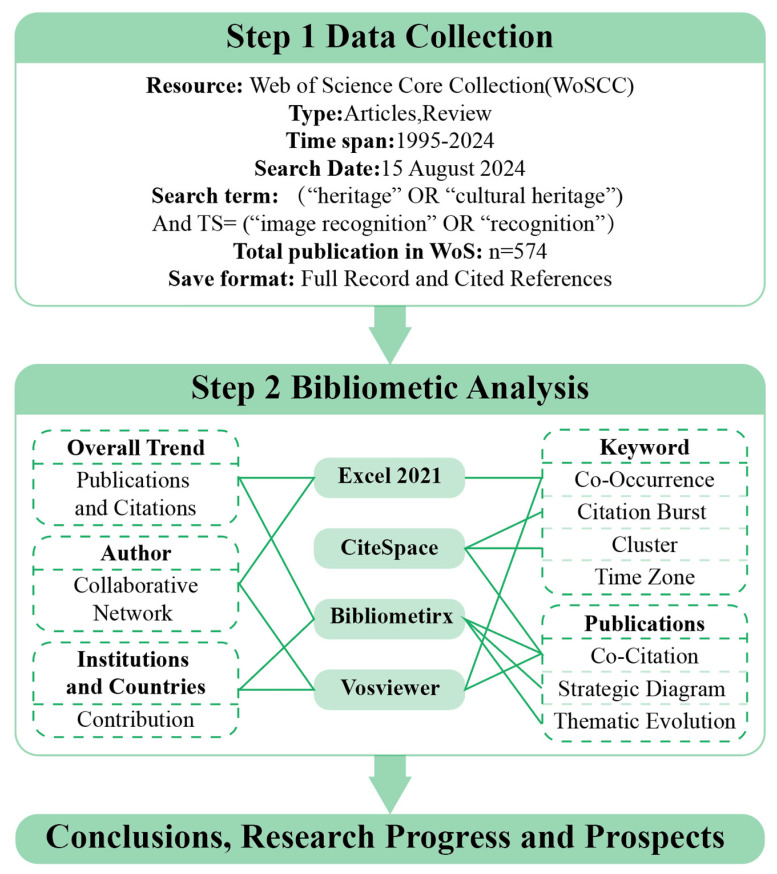
Research framework.

The data analysis followed a structured process. First, we imported the dataset into Bibliometrix software to analyze the annual trends in the number of publications and citations from 1995 to 2024 and used Excel 2021 for data analysis of the tables. Then, VOSviewer and CiteSpace software were used to analyze collaboration networks among authors, institutions, countries, and co-citations. Finally, we employed CiteSpace and Bibliometrix for keyword co-occurrence clustering, thematic evolution analysis, and burst term detection to identify research hotspots and trends in the field of image recognition in cultural heritage. The specific workflow is illustrated in Figure 1. By employing these bibliometric methods, this study provides a comprehensive overview of the research landscape, identifies critical research fronts, and highlights emerging trends in the application of image recognition techniques in cultural heritage.

## 3. Results

### 3.1. Overall Trend of Publications and Citations

**Figure 2:**
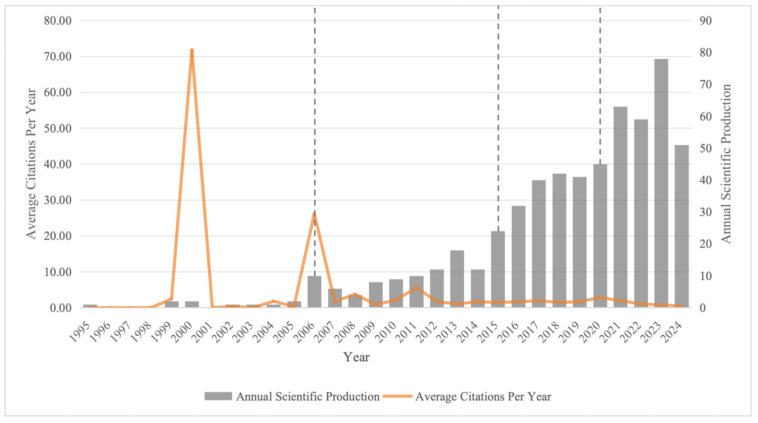
Overall trend of publications and average citations. Analysis performed using Bibliometrix.

Annual trend of publications and average citations per article on research on cultural heritage image recognition in the Web of Science Core Collection (WoSCC) database during 1995–2024, as shown in Figure 2.

Except for the years 1996 to 1998 and 2001, the number of papers on cultural heritage image recognition has generally shown an upward trend. Based on the number of papers and growth rates, this period can be divided into four phases: the nascent phase (1995–2005), the initial development phase (2006–2014), the steady growth phase (2015–2019), and the rapid growth phase (2020–2024).

Nascent Phase (1995–2005): Research in this field was still relatively sparse, with a slow annual growth rate in the number of papers. In 1994, the World Heritage Committee launched the Global Strategy for a Representative, Balanced, and Credible World Heritage List [53] to include more diverse and underrepresented heritage sites. This strategy expanded the scope of cultural heritage research and promoted the adoption of technologies such as image recognition after 1995 to better document, analyze, and manage cultural heritage. In 1999, the ICHIM 99 conference focused on themes such as multimedia and interactive technologies, virtual reality, digital archiving, and enhanced user experiences in cultural heritage management [54], significantly influencing the field of image recognition in cultural heritage by integrating advanced digital methods, which contributed to the peak in average citations per article in 2000 as these innovations spurred further research and cross-disciplinary interest.

Initial Development Phase (2006–2014): The number of papers increased overall compared to the previous phase. In 2006, a joint conference by CIPA, ISPRS, UNESCO, ICOMOS, VAST, Eurographics, and EPOCH in Cyprus focused on using high-resolution photogrammetry, laser scanning, 3D modeling, image processing, and virtual displays for digital recording, preservation, and interactive presentation of cultural heritage images [55], driving advancements in image recognition research in this field and contributing to a second peak in average citations per article in 2006.

Steady Growth Phase (2015–2019): The number of papers continued to grow steadily, with the average citation rate remaining at a relatively low level. Digital Heritage 2015 [56], EuroMed 2016 [57], EG GCH 2018, and 2019 [58,59] contributed to the steady growth in publications in the field of image recognition in cultural heritage from 2015 to 2019 by focusing on the application of computer vision and image recognition techniques, such as object detection and image classification, for the automatic analysis and preservation of cultural heritage images.

Rapid Growth Phase (2020–2024): There was a significant increase in the number of papers, with 2023 reaching the highest peak. CVPR 2022 [60], EG GCH 2021 [61], 2022 [62,63], and 2023 [64] have focused on the application of deep learning-based techniques for artifact image recognition, classification, and segmentation in cultural heritage preservation, driving rapid growth in this field and resulting in a record-high 78 publications in 2023.

### 3.2. Analysis of the Contribution of Authors, Institutions, and Countries

**Figure 3:**
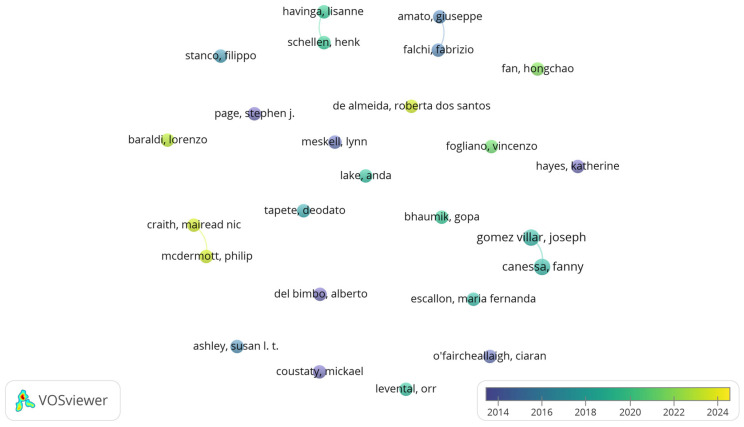
Co-authorship network map, analysis was performed using Vosviewer.

Collaboration networks among authors, institutions, and countries are primarily used to analyze the co-authorship of published articles [65], which helps to identify the main contributors in a research field and their collaboration patterns [66]. In the co-authorship analysis, 25 authors with the strongest links were selected from a total of 1368 authors, representing 21 different clusters. These authors appeared as nodes in at least two or more publications, showing 4 links with a total link strength of 9, indicating relatively low strength, as shown in Figure 3. This suggests that research in this field is conducted by multiple independent groups, reflecting a diverse range of research directions but also highlighting the potential for increased collaboration.

**Figure 4:**
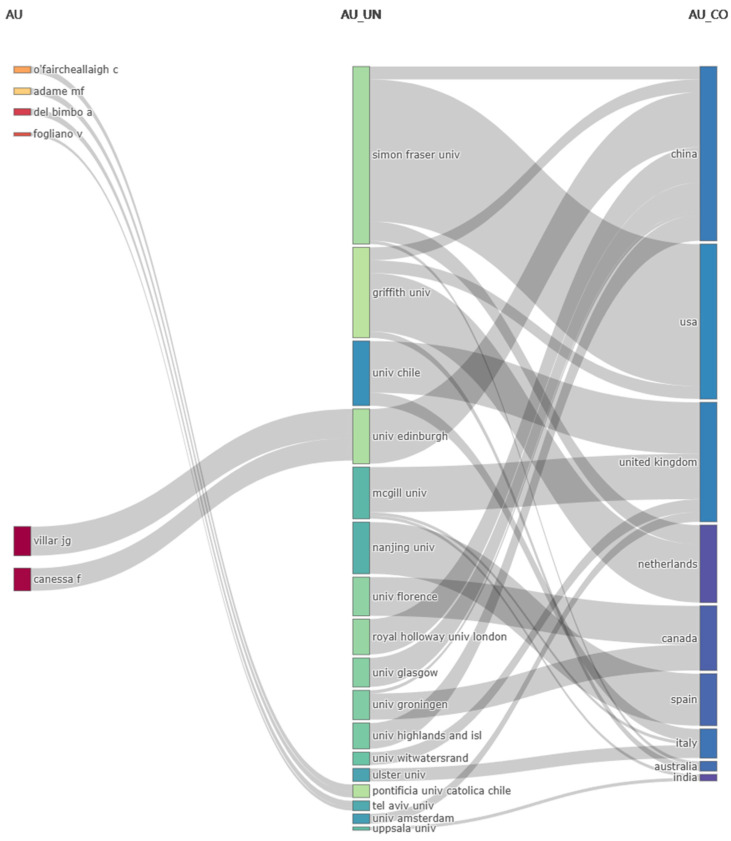
Affiliation relationships between authors and countries. Tree-field plot of affiliations (items 17), authors (items 6), and countries (items 9), produced using Bibliometrix.

A Sankey diagram visually represents the contributions and relationships among 6 prominent authors, their 17 affiliated institutions, and 9 countries in cultural heritage research [67] (Figure 4). The width of the connections reflects the level of collaboration or contribution between these entities [68], highlighting strong connections between Simon Fraser University and China, as well as between Griffith University and the USA, demonstrating global collaboration in cultural heritage research.

**Figure 5:**
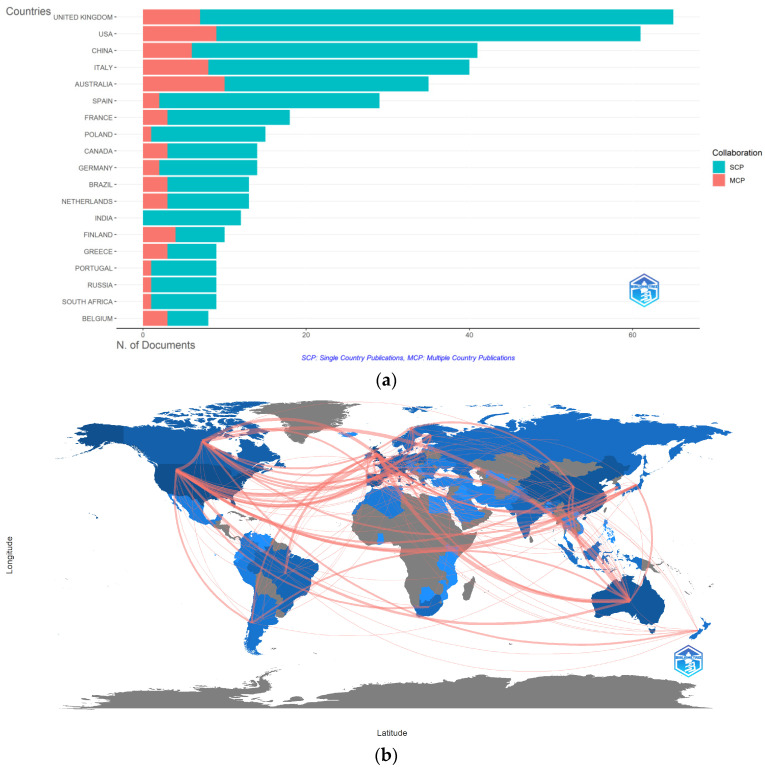
Country Analysis. (**a**) Corresponding Author’s Countries. (**b**) Country Collaboration Map. Analysis performed using Bibliometrix.

The analysis of the top 20 ranked publications by output shows that the United Kingdom leads with around 65 publications (Figure 5a). In most countries, single-country publications (SCPs) are significantly higher than multiple-country publications (MCP) [69], indicating that research is predominantly conducted by domestic teams rather than through international collaboration.

The country collaboration map displays the distribution of publications and collaborations between countries or regions worldwide [70], as shown in Figure 5b. European and American countries dominate scientific collaboration, while Asian countries’ influence is rising. The UK has the most international partners (33 countries), though cooperation with each is limited. Darker map colors indicate more publications, with the US, UK, Germany, China, and Australia contributing the most. Thicker lines represent stronger collaborations, such as between the US and UK and the UK and Australia, reflecting the globalization of scientific research.

### 3.3. Co-Citation Analysis of Publications

**Figure 6:**
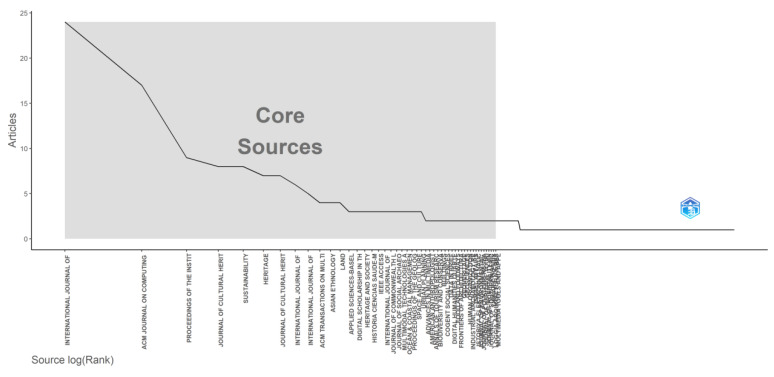
Core Sources by Bradford’s Law. Analysis performed using Bibliometrix.

By applying the principles of Bradford’s Law [71], Bibliometrix can help researchers identify the most active and influential journals within their dataset, thereby helping to determine the core journals in a specific field [72] and showing the distribution of journal articles in the field of image recognition in cultural heritage, as shown in Figure 6. Among these, the international journal of heritage has published the most articles, with around 25 articles, making it one of the most active journals in this field. The core journals mainly focus on topics such as image recognition and computer vision technologies, cultural heritage conservation and management, and interdisciplinary research and applications. It is evident that research in the field of “image recognition in cultural heritage” is highly interdisciplinary, combining aspects of technology development, cultural conservation, sustainability, and social science research.

**Figure 7:**
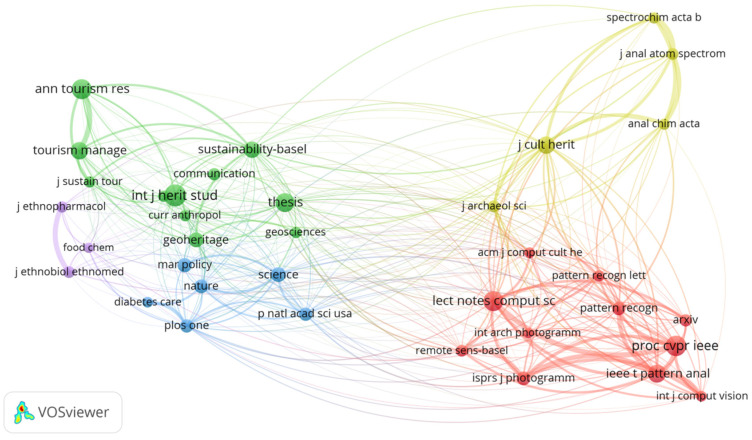
Publications co-citation network, analysis performed using Vosviewer.

The publication co-citation network is shown in Figure 7. With the minimum citation threshold set at 35, a total of 36 publications (nodes) met this criterion, where the size of each node represents the number of citations received by that publication. Co-citation links between publications are displayed as lines, with a total of 354 links.

Publications related to the field of image recognition in cultural heritage are divided into five clusters based on their citation frequency, with each cluster represented by a different color, indicating similar research topics or directions, as shown in Table 1. Notably, Cluster 1 and Cluster 2 are the largest, suggesting that these areas have attracted more substantial academic attention, whereas the smaller clusters, like Cluster 5, indicate emerging or more niche research topics.

**Figure 8:**
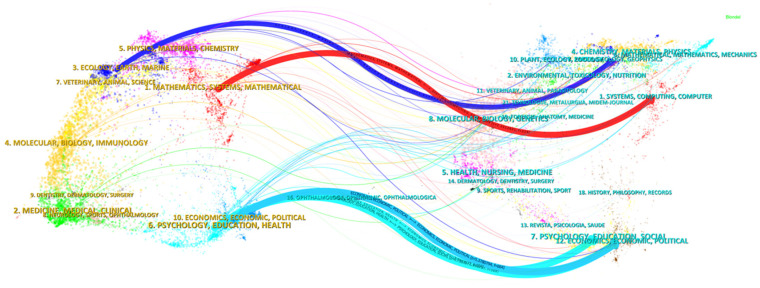
The dual-map overlay for related publications and analysis performed using CiteSpace.

The dual-map overlay is used to display the citation structure and disciplinary distribution of publications in the field of image recognition in cultural heritage research. Each color represents a different discipline to which a journal belongs, and the curves indicate citation paths [73]. The thicker the curve, the more frequent the cross-disciplinary citations, reflecting a close connection between the two disciplines. The map on the left is generated by mapping citing publications [69], representing the distribution of publications in the “cultural heritage image recognition” field. The map on the right is generated by the cited publications in the bottom figure [69], showing the disciplinary distribution of the references from the left map’s publications, as shown in Figure 8. The main disciplines and citation paths with thicker curves are shown in Table 2.

From Path One, it can be seen that there is a citation relationship between mathematics, systems science, and computer science in the field of image recognition in cultural heritage. Mathematical theories and systems methods provide theoretical support for the development of algorithms and models in the field of computer science, particularly in areas such as image processing, computer vision, and deep learning. Image recognition algorithms in computer vision, such as deep learning technology [74] and intelligence technology [75], often rely on mathematical models and systems theories for optimization and improvement.

### 3.4. Analysis of Hotspots and Trends

#### 3.4.1. Detection of Keyword Co-Occurrence

Keywords from titles and abstracts related to the research field of image recognition in cultural heritage were extracted to perform a co-occurrence analysis, visualizing the relationships between keywords and topics [76], identifying frequently studied topics, and detecting shifts in research focus over time.

**Figure 9:**
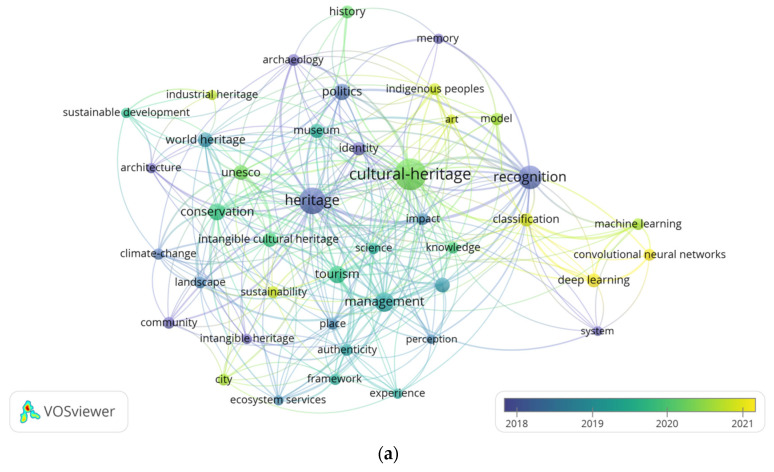

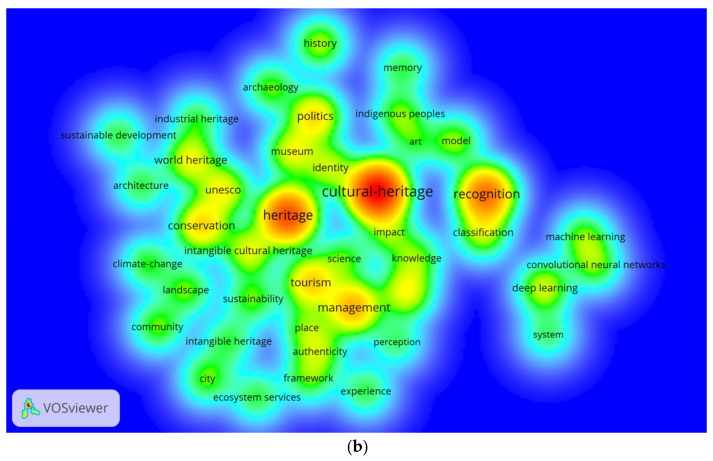
Keyword Co-Occurrence Analysis. (**a**) Keywords co-occurrence network. (**b**) Keywords Density Visualization. Analysis performed using VOSviewer.

In the Keywords co-occurrence network, each node represents a keyword, and the larger the node, the higher the frequency of the keyword’s occurrence [77]. The “co-occurrence” file type is selected, with the unit set to “all keywords”, as shown in Figure 9a. By setting a threshold of 7 occurrences for keywords, only 42 out of the initial 2984 keywords met this criterion. In the Keywords Density Visualization, areas with higher density, particularly those centered on keywords like ‘digital preservation’, ‘deep learning’, and ‘cultural heritage’, indicate focal points of current research, as shown in Figure 9b. The progression from cool to warm colors represents an increase in keyword frequency [52], reflecting the research focus in this field on how to use image recognition technology for the preservation and management of cultural heritage. Furthermore, clusters featuring keywords like ‘sustainability’ and ‘environment’ suggest an emerging interdisciplinary trend, where image recognition technologies are being explored for their potential to address environmental challenges in heritage preservation.

The top 10 keywords with the strongest links are shown in Table 3 and highlight key areas of focus in the field of cultural heritage image recognition. The Core keywords “cultural-heritage”, “heritage”, and “recognition” form the foundation of this research, underscoring its primary focus on heritage preservation and image recognition techniques. Meanwhile, high-frequency keywords like “management”, “tourism”, “conservation”, and “politics” reveal specific applied areas within the field. The high frequency of “management” reflects a growing focus on the administrative and operational aspects of cultural heritage preservation, where image recognition technologies are applied to improve the digital management, monitoring, and protection of heritage assets [78,79]. Similarly, “conservation” underscores the importance of using advanced technologies, such as AI and deep learning, to detect damage and support the sustainable preservation of cultural artifacts and sites [80,81]. At the same time, “tourism”, as another high-frequency keyword, highlights the practical value of image recognition technology for cultural heritage tourism, such as in enhancing visitors’ knowledge and digitizing landscape heritage [82,83]. Additionally, “politics” points to the role of political frameworks, international cooperation, and policy-making in heritage management, reflecting broader social and policy research that examines how political factors influence the conservation and promotion of cultural heritage [84,85]. Together, these keywords illustrate how the field extends beyond technical innovations, engaging with interdisciplinary themes such as site management, sustainable tourism, conservation strategies, and the political dynamics that shape heritage preservation.

#### 3.4.2. Cluster Analysis

**Figure 10:**
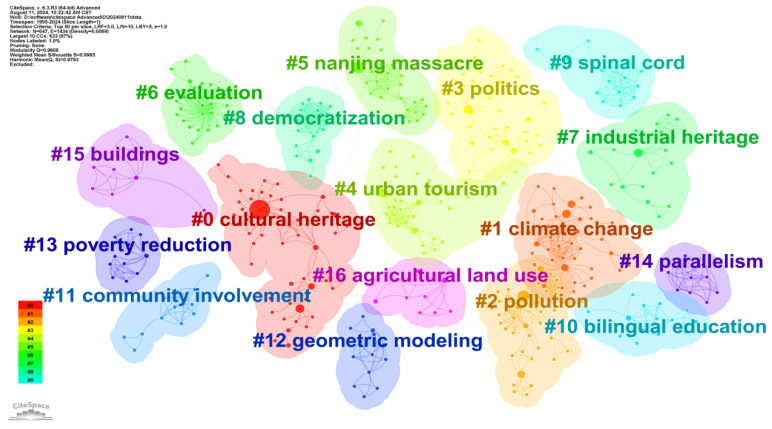
Keyword cluster map. Analysis performed using CiteSpace.

The Keyword cluster map analysis considered all keywords and divided them into several clusters [70], with each cluster having its own color and label. The label numbers, arranged from smallest to largest, represent the clusters in order from largest to smallest. The smaller the number, the more members the cluster contains. Using the g-index (k = 10) as the selection criterion, the resulting network contains 17 clusters, as shown in Figure 10. The Modularity Q is 0.960, indicating good clustering quality, with nodes within each cluster being closely connected, suggesting that different research topics (clusters) are relatively independent. The Weighted Mean Silhouette score is 0.9985, indicating a very clear clustering structure, with high similarity among keywords within clusters and very distinct differences between different clusters.

As shown in Figure 10, the top five clusters are Cultural Heritage, Climate Change, Pollution, Politics, and Urban Tourism, indicating the main research directions and hotspots in the field of image recognition in cultural heritage. These involve topics such as cultural heritage preservation and management, the impact of climate change and pollution, politics and policy, and urban tourism. The keywords that best represent the theme of each cluster are listed in Table 4. Based on their average formation time, the most recently formed cluster is Cultural Heritage, Conversely, Pollution, which was formed earlier.

**Figure 11:**
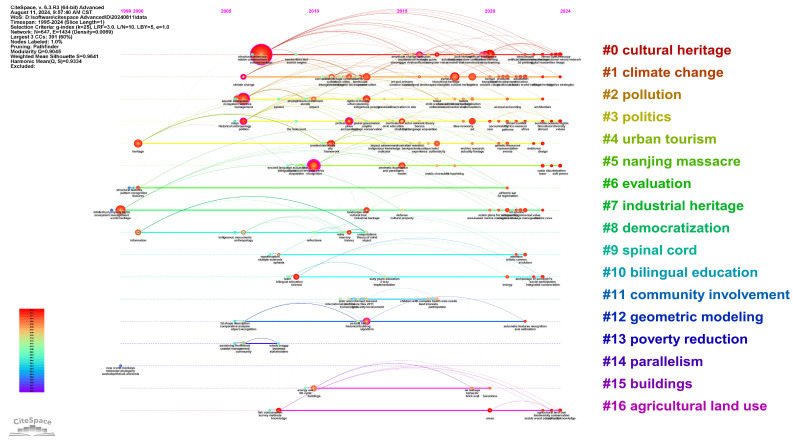
The timeline view of keywords.

Based on Figure 11, the evolution of citation clusters can be observed through the timeline graph [86]. Research published between 1980 and 2022 was selected for analysis, with a time slice length of 1 year, selecting up to the top 25 highly cited publications for each period. Clusters #0, #1, #3, and #5 have remained active over a long period, underscoring their representation of enduring research topics. The largest circle appears in the largest cluster, Cultural Heritage, indicating that during the 2004–2010 period, the keyword “cultural heritage” had the highest number of publications and was closely associated with “structural behaviour”, highlighting significant attention to the conservation and restoration of cultural heritage buildings during this period [87]. The short connection line between “built heritage” and “artificial intelligence” suggests a strong collaboration between them, such as the use of artificial intelligence technology to quickly obtain key information on the geometry, materials, and structural characteristics of buildings damaged after earthquakes, enabling the protection and prevention of built heritage [88].

Among them, Cultural Heritage is the largest cluster, containing the most keywords, with 52 in total, and a Silhouette value of 0.973. This indicates that the research focus of this cluster is highly consistent and concentrated within the field of image recognition in cultural heritage. Most of the related literature was published around 2019. From the Top Terms, the representative keywords mainly include “cultural heritage”, “deep learning”, “machine learning”, “computer vision”, and “digital humanities”, indicating that the main research direction of this cluster is the combination of cultural heritage with deep learning, machine learning, and computer vision technologies, including exploratory methods for digital excavation and information annotation reconstruction of cultural heritage [89,90] such as calligraphy character recognition [91], object detection in art images [37], and handwriting recognition for ancient manuscripts [92]. Additionally, the automatic recognition of buildings in urban heritage images [89] and the application of these emerging technologies in digital humanities [93] are also important research directions in this cluster. From the Terms (mutual information), keywords such as “braille alphabet”, “information accessibility”, “seismic”, “chemometrics”, and “acoustics” are representative keywords with similar characteristics in this cluster, all having a mutual information value of 1.19. This suggests that the cluster may involve diverse themes in cultural heritage conservation, such as promoting braille accessibility through image recognition technology [94], analyzing multivariate data of artifacts through chemometrics [95], or protecting ancient buildings through acoustic monitoring [96].

According to Table 5, among the top 10 keywords with bursts starting from 2020 to the present, “cultural heritage” has the highest burst strength, followed by “deep learning” and “world heritage”. The word with the highest centrality is “recognition”, followed by “cultural heritage”. This indicates that “cultural heritage” is not only a recent research hotspot but also serves as a connector and bridge within the entire research network. “Recognition” reflects its critical role in tasks such as cultural heritage image analysis and classification using deep learning and artificial intelligence technologies. As a methodological keyword, it establishes significant connections between multiple related research topics [89,97]. Intelligent image recognition technology, as a methodological tool, plays a crucial role in analyzing and classifying cultural heritage images by improving recognition accuracy, reducing training time, and minimizing information loss. It also facilitates the digital preservation and dissemination of cultural heritage [98]. Additionally, the high burst of “deep learning” demonstrates its important role in driving technological innovation in the field of cultural heritage preservation, particularly in areas such as image recognition, data analysis, and optimization of conservation strategies [74,99,100].

#### 3.4.3. Strongest Citation Bursts

**Figure 12:**
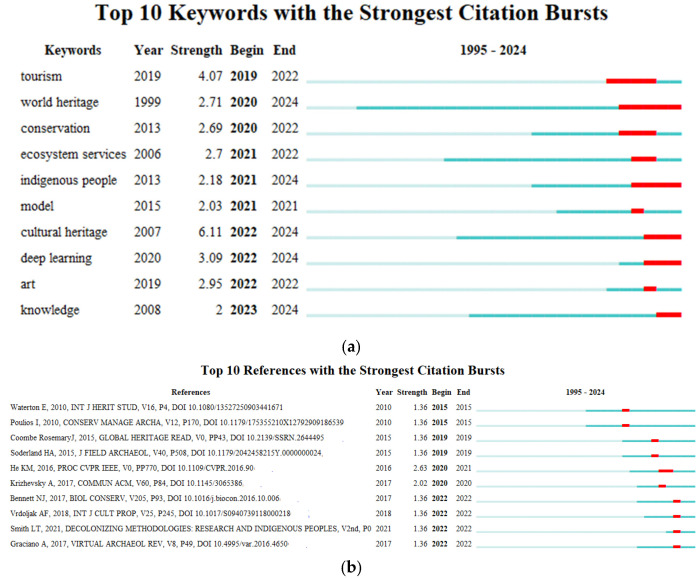
Citation burst analysis. (**a**) The top ten keywords with the strongest citation bursts. (**b**) The top 10 references with the strongest citation bursts. Analysis performed using CiteSpace.

Citation burst analysis was conducted to highlight articles that have attracted exceptional attention within short periods, indicating emerging trends in this field [101], as shown in Figure 12.

In Figure 12a, the Strength value of each keyword represents the rate or magnitude of increase in citations for that keyword over a certain period. “Cultural heritage” has the highest burst strength (6.11), indicating a significant increase in citations from 2007 to 2024, making it a sustained hot topic in the research field. The Begin and End times of each keyword indicate the start and end years of its citation burst. For example, the citation burst for “tourism” occurred from 2019 to 2022, suggesting that this keyword received significant academic attention during this period. “World heritage” (1999–2024) and “cultural heritage” (2007–2024) show very long citation burst periods, indicating that these keywords have maintained high levels of attention in cultural heritage research over the long term. Keywords such as “deep learning” (2020–2024) and “knowledge” (2023–2024) show recent bursts, reflecting their rapid rise in recent years and suggesting that these technologies or topics are becoming emerging hotspots, especially in applying modern artificial intelligence and deep learning techniques to solve cultural heritage recognition problems, such as those involving historical document images and traditional Chinese paintings [102,103]. “Ecosystem services” (2021–2024) and “indigenous people” (2021–2024) also show strong citation bursts, indicating increasing interest among researchers in using AI technologies to support ecosystem services for cultural heritage [75] and exploring the relationship between heritage and indigenous peoples [104,105].

Figure 12b shows the top 10 publications in the field of image recognition in cultural heritage with the fastest growth in citation counts. The co-citation strength of He KM, 2016, is 2.63, the highest on the list, indicating that Deep Residual Learning for Image Recognition is considered one of the most advanced and effective methods for image recognition [106], which has significantly increased in influence between 2020 and 2021 due to its ability to help address image recognition challenges in cultural heritage data. The citation burst period for each publication indicates the years during which it received significant citation attention. For example, references with a citation burst in 2022 highlight that topics such as cultural heritage digitization [107], methods for heritage preservation of Indigenous Peoples [108], and improving heritage conservation through social sciences [109] received a great deal of academic attention that year.

#### 3.4.4. Thematic Evolution of Publications

**Figure 13:**
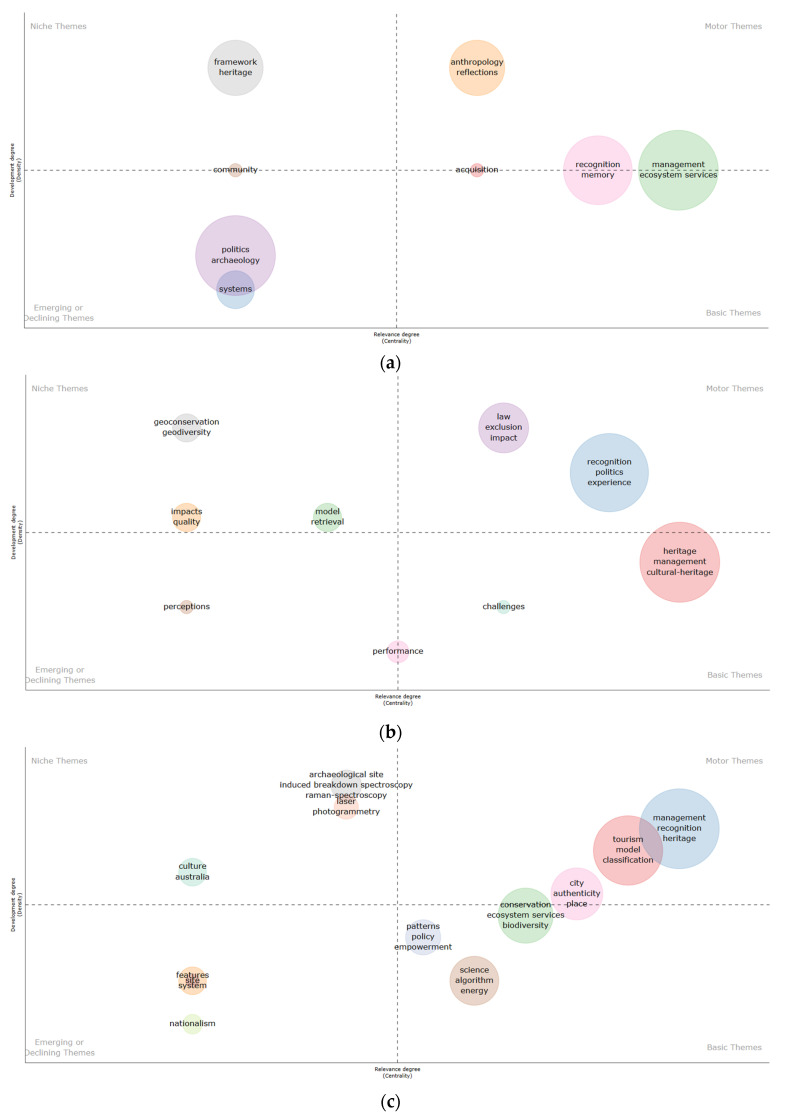
Keywords thematic map. (**a**) 2006–2014. (**b**) 2015–2019. (**c**) 2020–2024. Analysis performed using Bibliometrix.

Thematic evolution analysis is used to track how research themes in the field of image recognition in cultural heritage have developed and transformed over time, providing insights into future directions. The thematic map reveals key themes and patterns through word network analysis [110], while thematic analysis, as a qualitative method, explores patterns in the data and uncovers interrelationships between phenomena. Based on centrality (the degree of a theme’s connection to other clusters) and density (the cohesion of keywords within a cluster), research themes are divided into four quadrants in a strategic diagram [73,111], displaying research hotspots, as shown in Figure 13. Each circle in the diagram represents a theme, and its size depends on the frequency of the keywords [69]. The top-right (Motor Themes) quadrant represents driving topics that are well-developed and are crucial to the field of Image Recognition in Cultural Heritage. The top-left (Niche Themes) quadrant is highly specialized and relatively isolated, with fewer researchers focused on them. The bottom-left (Emerging or Declining Themes) quadrants are relatively underdeveloped and typically follow the thematic directions of other quadrants. The bottom-right (Basic Themes) quadrant in the bottom right have high centrality and low density that may be potential research hotspots.

Based on the number and growth rate of publications related to the field of Image Recognition in Cultural Heritage, the research period since the emergence of this field can be divided into four phases. Among them, the number of publications from 1995 to 2005 was relatively low; therefore, the thematic evolution chart mainly analyzes the following three sub-periods: (a) 2006–2014, (b) 2015–2019, and (c) 2020–2024.

From Figure 13a, it can be seen that in the first sub-period (2006–2014), “management”, “anthropology”, “reflections”, and “recognition” were the driving topic. “Framework” was the isolated topic. “Politics”, “archaeology”, and “systems” represent emerging or declining themes, while topics with development potential included “ecosystem services” and “memory”. Figure 13b shows the second sub-period (2015–2019); “law”, “exclusion”, “impact”, “recognition”, “politics”, and “experience” were the driving topics. “Geoconservation”, “geodiversity”, “impacts”, “model”, “quality”, and “retrieval” were the isolated topics. “Perceptions” represent emerging or declining themes, while topics with development potential included “heritage”, “management”, “cultural-heritage”, and “challenges”. Figure 13c shows the third sub-period (2015–2022); “management”, “recognition”, “tourism”, “heritage”, “model”, “classification”, “city”, and “authenticity” were the driving topics. “Archaeological site”, “induced breakdown spectroscopy”, “raman-spectroscopy”, “laser”, and “photogrammetry” were the isolated topics. “Features”, “site”, “system”, and “nationalism” represent emerging or declining themes, while topics with development potential included “ecosystem services”, “patterns”, “biodiversity”, “policy”, “empowerment”, “science”, “algorithm”, and “energy”. These observations help in understanding the evolution of research themes over different periods and provide researchers with clues to predict potential research hotspots.

**Figure 14:**
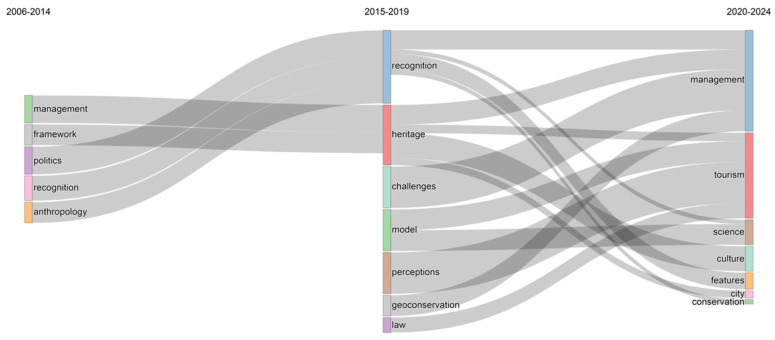
Thematic evolution chart. Analysis performed using Bibliometrix.

The thematic evolution of Image Recognition in Cultural Heritage related publications from 2006 to 2024, as shown in Figure 14. In the first sub-period, “politics”, “recognition”, and “anthropology” merged into “heritage” in the next sub-period and further split into “culture”, “features”, and “city” in the third sub-period. Meanwhile, they also merged with new themes from the second sub-period, “challenges” and “geoconservation”, to form “management”. “Management” and “framework” from the first sub-period merged into “recognition” in the next sub-period and then further split into “science”, “features”, and “conservation” in the third sub-period, with some of the split themes merging into “management”. The newly emerged keyword “model” from the second sub-period split into “tourism” and “science” in the next phase, while “perceptions” and “law” merged into “tourism”. The evolution of these themes shows a continual shift in research focus, gradually expanding from policy and management to specific application areas such as deep learning and tourism management, while also integrating multidisciplinary directions such as environmental science, social policy, and technological optimization.

## 4. Discussion

### 4.1. Keyworks and Theme Evolutionary Analysis

This paper conducts a bibliometric analysis of the literature on image recognition in cultural heritage from 1995 to 2024, revealing the research development trajectory, hot topics, and future research trends in this field. The bibliometric analysis of image recognition in cultural heritage, based on keyword co-occurrence and clustering, reveals clear research themes such as ‘cultural heritage management’, ‘tourism’, and ‘conservation’ [112]. The frequent appearance of keywords like ‘deep learning’ and ‘AI’ reflects the growing importance of these technologies in addressing challenges related to heritage preservation and digital documentation [113,114]. Furthermore, the clustering of keywords such as ‘politics’ and ‘sustainability’ indicates an increasing interdisciplinary focus, integrating social, political, and environmental dimensions into the technical aspects of cultural heritage research [115].

Since 1995, research in the field of image recognition in cultural heritage has undergone significant development and evolution. The starting point of this research can be traced back to the response to the need for cultural heritage protection, particularly in the face of threats such as environmental degradation, urban development, and war conflicts on a global scale [1], as well as the formulation of the World Heritage “Global Strategy” [116]. From 1995 to 2005, research themes mainly focused on methodological and theoretical studies related to the framework and management of cultural heritage preservation, especially the initial attempts to apply image recognition technology to analyze and monitor cultural heritage [117]. The year 2000 had the highest number of citations, driven by key organizations and policies promoting the application of image recognition technology in the field of cultural heritage. With technological advancements and the acceleration of digital transformation, there was a rise of heritage as a global and local phenomenon [116]. After 2006, research gradually shifted toward practical applications, particularly in management and conservation strategies. During this period, core keywords such as heritage (cluster 4), features (cluster 6), pattern recognition (cluster 6), world heritage (cluster 7), and information (cluster 8) emerged. Among them, world heritage had the strongest citation burst, indicating that it was a central theme driven by global initiatives to protect and digitize cultural heritage [118]. Despite the technical innovations in pattern recognition (which peaked between 2000–2009), most of these early themes continued to remain active well into the 2020s, reflecting their enduring relevance [81,119]. With technological advancements and the acceleration of digital transformation [57], cultural heritage began to rise as both a global and local phenomenon, prompting further methodological exploration.

In the three sub-periods, research themes in the field of image recognition in cultural heritage underwent significant changes. In the first sub-period (2006–2014), research topics in the field of Image Recognition in Cultural Heritage mainly focused on the initial integration of image recognition technology with cultural heritage, emphasizing the exploration of fundamental concepts and application scenarios. The core research topics involve reflections on cultural anthropology and the application of image recognition technology, reflecting the construction of the theoretical foundation in the early stages of research. This period was marked by key themes such as cultural heritage (cluster 0), management (cluster 2), recognition (cluster 5), politics (cluster 3), indigenous people (cluster 2), conservation (cluster 1), and ecosystem services (cluster 2). The keywords conservation and ecosystem services had the strongest citation bursts, highlighting their growing importance. This period saw the expansion of research beyond the technical aspects of image recognition, with management playing a central role in the development of frameworks for heritage site administration [120,121]. Additionally, politics and archaeology signaled the growing interest in the social and political dimensions of heritage preservation [84,122,123], as image recognition technologies began to intersect with broader cultural and ecological concerns [124]. Keywords like ecosystem services reflected a more interdisciplinary approach, incorporating environmental science into heritage management strategies [125,126].

In the second sub-period (2015–2019), research themes became significantly more diverse, indicating that the application of image recognition in the field of cultural heritage was gradually maturing during this period and further expanding into diverse directions, including legal, political, and empirical perspectives. This stage of research tends to combine legal and social impacts with technological development and application, with significant research efforts directed toward “heritage”, “management”, and “cultural-heritage”, indicating a shift in focus from theory to application. Important keywords during this period included intangible cultural heritage (cluster 1), tourism (cluster 1), model (cluster 5), art (cluster 5), and classification (cluster 0). These keywords, which burst onto the research scene after 2019, reflect the increasing importance of non-material aspects of heritage, such as traditions and practices, as well as the application of advanced models for heritage analysis. Notably, tourism had the strongest citation burst, illustrating the growing integration of image recognition technologies into the tourism sector to enhance visitor experiences and support heritage site sustainability [127,128]. The prominence of intangible cultural heritage indicates the extension of image recognition technology from physical artifacts to the preservation of cultural knowledge and practices [129,130], while model and classification point to the continued development of new methods for organizing and analyzing heritage data [131,132].

In the third sub-period (2020–2024), research themes became more refined and diversified, further strengthening the integration of technological applications with the field of cultural heritage. The driving topic has evolved from further optimization of management and recognition technologies to integration with tourism and urban planning. This reflects a high level of attention to the application of image recognition technology in diverse cultural heritage scenarios, especially in areas such as data management, classification, and authenticity detection. More specialized technical research and application contexts focus mainly on specific scientific methods and technical tools. At the same time, future research directions in the field of image recognition in cultural heritage will further integrate multidisciplinary content such as environmental science, algorithm optimization, social policy, and energy management, presenting significant research potential and prospects. Important keywords during this phase include deep learning (cluster 0), machine learning (cluster 0), adaptive reuse (cluster 7), artificial intelligence (cluster 0), heritage management (cluster 7), traditional food (cluster 1), and UNESCO world heritage (cluster 1). Deep learning had the strongest citation burst, highlighting its critical role in advancing image recognition techniques for heritage preservation [133,134]. The adoption of AI and machine learning technologies in heritage management has led to breakthroughs in automating the recognition, classification, and restoration of heritage objects [135,136]. Furthermore, keywords such as adaptive reuse and heritage management reflect the growing interest in using technology to optimize the sustainable reuse of heritage sites and structures [134,137,138]. As this period progressed, the research focus extended to more specific scientific methods and tools, including AI-driven models for detecting authenticity, managing heritage data, and promoting conservation efforts [139,140,141].

Overall, “cultural heritage” has consistently been the core theme in this field. As research has deepened, it has gradually expanded from studies on broader contexts, such as “systems” and “politics” to technical and managerial aspects, including “deep learning” and “management”. The research focus has progressively moved toward more specific digital applications and interdisciplinary integration, encompassing not only the preservation of physical artifacts but also intangible heritage, tourism, environmental sustainability, and political considerations. Researchers are increasingly exploring how artificial intelligence and machine learning technologies can optimize ecological monitoring, energy management, and social policy at cultural heritage sites, while also promoting public participation and community empowerment. This shift has enhanced the accuracy and efficiency of heritage recognition and preservation, adding depth and diversity to the field. Looking forward, future research directions will likely continue to integrate multidisciplinary content, including environmental science, algorithm optimization, social policy, and energy management, presenting promising research potential in both theoretical and applied areas of cultural heritage preservation.

### 4.2. Affiliation Relationships Analysis

From the affiliation relationships between authors and countries, it can be observed that academic contributions in the field of cultural heritage research exhibit a clear pattern of international cooperation. First, some countries and institutions play a key role in promoting cross-national academic collaboration and knowledge exchange. Simon Fraser University (Canada), Griffith University (Australia), and the University of Chile (Chile) are among the main institutions shown in the diagram, with strong collaborative relationships with multiple countries and other institutions. Their research contributions focus on cross-national collaboration, reflecting the trend of internationalized research in the field of cultural heritage. Second, China and the United Kingdom demonstrate broader patterns of cooperation and contribution. They are connected to multiple institutions and authors, indicating their extensive involvement and significant status in the field of cultural heritage research.

Among the highly contributing institutions, authors from CNR explore diverse research directions in cultural heritage, including the use of AI-based solutions for efficient masonry annotation in architectural heritage, visual search systems for ancient inscriptions, advanced kNN-based methods for monument recognition, detecting fake online reviews related to heritage tourism using language models, and applying near-infrared spectroscopy for non-destructive analysis of traditional wooden flooring [100,142,143,144,145]. Each of these research directions demonstrates innovative applications of technology to enhance the preservation, recognition, and analysis of cultural heritage. Authors from the University of Florence explore advanced digital technologies in cultural heritage, focusing on content-based retrieval of 3D models for robust object recognition and developing a smart audio guide system that utilizes computer vision and deep learning to enhance visitor interactions in museum settings [146,147]. The University of Edinburgh’s latest research direction focuses on the impact of Handwritten Text Recognition (HTR) technology on the information environment and its multi-domain applications [148]. Through a literature review, the institution systematically outlined, for the first time, the diversity of application areas for HTR technology, highlighting its significant role in accelerating text transcription and analysis. Authors from the University of Groningen focus on developing a search engine for digitized handwritten historical documents, utilizing artificial intelligence and human-computer interaction to optimize search results and address the complex challenge of recognizing specific information, such as names of people and places. [149]

The most cited author during this period, Del Bimbo, Alberto, focused on advancing computer vision and machine learning techniques, particularly in the domain of cultural heritage image recognition. Between 2006 and 2014, he contributed to the development of content-based retrieval of 3D models, enhancing the analysis and digital reconstruction of heritage artifacts [147]. He also pioneered the creation of a smart audio guide system for museums, leveraging CNNs for real-time object recognition [146], which improved visitor interaction and accessibility to cultural heritage assets in museum settings. The second most cited author is Amato, Giuseppe, who advanced methods for cultural heritage image recognition between 2015 and 2019, demonstrating that novel kNN-based techniques and combined Fisher Vector (FV) and Convolutional Neural Network (CNN) features significantly enhance the recognition accuracy of monuments, landmarks, and ancient inscriptions [143,144].

### 4.3. Advances in Technology and Methods

With technological advancements, particularly the rapid development of deep learning and algorithm optimization, research methods in cultural heritage image recognition have evolved through distinct phases. Initially, from 1995 to 2005, pattern recognition and feature extraction techniques were employed [150], focusing on the identification of simple visual elements like shapes and textures to categorize cultural artifacts [151,152]. As research progressed from 2006 to 2014, the field witnessed the adoption of machine learning algorithms, such as spatial pyramid matching and boosted cascade classifiers [114,153], which enhanced the accuracy of object recognition in complex environments. From 2015 to 2019, deep learning frameworks—particularly Convolutional Neural Networks (CNNs)—emerged, automating feature extraction and improving the precision of cultural heritage analysis [6]. More recently, from 2020 to 2024, the integration of AI and IoT technologies has driven advancements across digital documentation, archaeological analysis, and interactive applications, enabling real-time monitoring and proactive heritage preservation.

From 1995 to 2005, image recognition in cultural heritage relied primarily on pattern recognition and feature extraction techniques, such as Scale-Invariant Feature Transform (SIFT) and Speeded-Up Robust Features (SURFs) [151]. These methods enabled more reliable recognition of key features, providing a foundational approach for analyzing cultural artifacts in complex and cluttered environments. Systems like content-based image retrieval (CBIR) were used to compare visual similarities between heritage objects, enabling the early stages of automated cultural heritage analysis [152]. However, these methods faced limitations, particularly in handling degraded or occluded images—a common challenge in cultural heritage settings. They also required high computational power and struggled to generalize across diverse artifacts. Toward the end of this phase, researchers began exploring machine learning algorithms such as AdaBoost and Support Vector Machines (SVMs) to improve object detection accuracy, automate classification, and enhance processing speed [150]. These developments collectively pushed the boundaries of image recognition capabilities, setting the stage for the future integration of deep learning and artificial intelligence in the preservation and analysis of cultural heritage.

Between 2006 and 2014, the field of image recognition in cultural heritage witnessed a transition toward more advanced machine-learning techniques, which improved both feature extraction and object classification. One notable development during this phase was the introduction of the spatial pyramid matching framework, which organized image features hierarchically, improving recognition accuracy for complex heritage contexts such as archaeological artifacts and historical sites [153], which led to higher accuracy in recognizing diverse image types. Random Forest classifiers further optimized object classification by combining visual and shape features, enhancing robustness against background clutter and improving accuracy on large datasets [154]. These models significantly reduced the challenges posed by background complexity, while also enhancing the processing of large datasets—a major advantage during this period.

However, despite these advancements, machine learning algorithms still face challenges. One key limitation was their difficulty in addressing the semantic gap in content-based image retrieval (CBIR) systems. This gap often led to mismatches between retrieval results and user expectations, particularly in heritage image databases where metadata were incomplete or unstructured. Moreover, these algorithms struggled with scaling across unstructured data typical in cultural heritage settings, where data could be highly fragmented or incomplete.

Despite advancements in machine learning algorithms during this period, initial challenges in addressing the semantic gap in content-based image retrieval (CBIR) systems persisted. This gap often led to mismatches between retrieval results and user expectations, particularly in heritage image databases where metadata were incomplete or unstructured. Scaling across unstructured data was another issue, as cultural heritage settings often presented highly fragmented or incomplete datasets. However, researchers began to address these limitations by developing multidimensional approaches that dynamically interacted with various visual features, improving the alignment of retrieval results with user expectations [124]. These advancements significantly improved the effectiveness of heritage image retrieval and facilitated more user-focused searches. This phase also marked the expansion of image recognition methods beyond modern object detection, showcasing their versatility in identifying historical artifacts and structures, thus improving heritage preservation efforts [155]. This improved the effectiveness of heritage image retrieval and facilitated more user-focused searches.

Innovative methods for recognizing distinct art styles also emerged during this period, with new techniques leveraging targeted visual cues such as color contrast and illumination to classify paintings more effectively [156]. Simultaneously, named-entity recognition techniques were developed to mine unstructured metadata, improving search and discovery functionalities in cultural heritage databases [157]. These methods allowed for more interactive exploration of archaeological sites, where semantic extraction was applied to tailor user experiences based on individual preferences and profiles [158]. Toward the end of this period, early advancements in deep learning began to influence the field. Multi-layer neural networks significantly enhanced the classification of visual styles across large datasets, showing great potential for broad applications in cultural heritage [159]. However, the computational costs and the need for large amounts of labeled data limited the adoption of these models by smaller heritage institutions during this period. These early **deep-learning** approaches paved the way for the more advanced AI-driven techniques that would dominate the subsequent phases of image recognition research.

Between 2015 and 2019, the field of cultural heritage image recognition experienced significant advancements through the integration of deep learning and other innovative technologies. Convolutional Neural Networks (CNNs) revolutionized the field by automating the feature extraction process, enabling more precise recognition of intricate details such as textures, carvings, and architectural elements [114]. These networks improved accuracy and speed by analyzing large datasets with multiple layers of abstraction, significantly outperforming previous machine-learning techniques. For instance, CNNs were successfully applied to the classification of historical artifacts and the analysis of ancient manuscripts, enhancing the efficiency of heritage documentation and restoration tasks [6]. This automation of feature extraction offered unprecedented accuracy in recognizing intricate details, contributing to remarkable success in documentation, restoration, and classification tasks in the cultural heritage domain. However, deep learning comes with challenges. These models require vast amounts of labeled data, which is often scarce in the cultural heritage domain. Additionally, deep learning models are computationally expensive and require substantial resources for training and deployment, posing barriers for smaller institutions lacking access to advanced hardware and large datasets. Despite these limitations, deep learning’s ability to handle large-scale datasets and improve recognition precision made it a key advancement during this period.

During this period, novel image-based recognition features that leveraged spatial structures were developed, leading to higher accuracy and efficiency in recognizing historical artifacts and objects [147,160]. Advanced Optical Character Recognition (OCR) methods were also enhanced to process degraded early modern texts, improving the accessibility of cultural heritage documents [161]. In the realm of ancient art analysis, multi-view local color features and deep learning techniques such as Deep Convolutional Neural Networks (DCNNs) were employed to classify artifacts and predict art styles, demonstrating improved precision and new insights for cultural heritage classification tasks [162]. Moreover, gesture recognition technology was explored to digitally annotate traditional cultural performances, bridging the gap between new media art and intangible cultural heritage preservation [163]. Real-time computer vision systems were introduced in interactive museum guides, enhancing user engagement with cultural artifacts through more immersive experiences [164]. Machine learning approaches were adapted to analyze typographical elements in historical documents and to restore and preserve degraded handwritten manuscripts using deep learning techniques [165,166]. Additionally, machine learning and citizen science approaches were integrated to detect and classify archaeological objects in remote sensing data, demonstrating the potential of automated detection systems in archaeological prospection [165,167,168]. These advancements collectively marked a transformative phase in the application of image recognition and deep learning technologies for cultural heritage analysis and preservation, opening new avenues for the study, documentation, and interactive engagement with cultural heritage assets.

From 2020 to 2024, the integration of artificial intelligence (AI) and Internet of Things (IoT) technologies further expanded the scope of image recognition in cultural heritage across several key areas, including digital documentation, archaeological analysis, heritage object recognition, and interactive applications. In digital documentation, CNNs enhanced image retrieval and recognition of heritage elements, supporting more accurate educational and preservation efforts [21]. Pattern recognition and AI techniques in a multidisciplinary context introduced new tools for heritage analysis and reconstruction, allowing for more comprehensive approaches to documenting and preserving cultural assets [169]. Additionally, IoT-based interactive environments provided curators with the means to create personalized experiences at heritage sites [140], enhancing engagement through real-time interaction.

In archaeological analysis, AI-powered models like U-Net were optimized for detecting subsurface features from remote sensing data [93,170], enabling non-invasive investigations of heritage sites. This integration of AI and IoT enabled real-time monitoring and proactive preservation of heritage sites, allowing curators and researchers to track environmental changes and the structural integrity of sites in real-time, thus preventing potential damage through early intervention. Generative Adversarial Networks (GANs) were also employed to restore damaged artifacts, such as artworks and buildings, reconstructing missing sections by learning from historical datasets, significantly improving restoration accuracy [171,172]. Furthermore, innovative image processing techniques identified surface materials like potsherds, improving survey efficiency and accelerating archaeological discovery [173]. New evaluation measures were also introduced to better assess machine learning-based object detection in remote sensing data, providing more reliable archaeological prospection results [174]. However, these technologies also come with significant challenges. AI and IoT technologies require substantial investment in infrastructure and technical expertise, which may be out of reach for many cultural heritage institutions. Additionally, there is a risk of over-reliance on technology for decision-making in heritage conservation, potentially leading to ethical concerns about the preservation process.

Furthermore, innovative image processing techniques identified surface materials like potsherds, improving survey efficiency and accelerating archaeological discovery. New evaluation measures were introduced to better assess machine learning-based object detection in remote sensing data, providing more reliable archaeological prospection results. Deep learning also enabled the automated extraction of georeferenced information from historical maps, aiding large-scale landscape reconstruction [175]. For heritage object recognition, AI models advanced the detection and classification of typographical elements in historical documents [176], facilitating more precise analysis of degraded manuscripts. CNNs were also adapted for recognizing cultural symbols [177] and cross-time registration of heritage objects [178], enhancing the accuracy of identifying artifacts across different historical periods. A new backend model supported Optical Character Recognition (OCR) and document retrieval, improving access to historical documents [179]. Advanced deep learning models achieved high accuracy in categorizing historical illustrations [180] and enabled non-invasive manuscript material analysis [181], providing new insights into cultural preservation.

Moreover, deep semantic binarization networks demonstrated effectiveness in restoring degraded heritage documents, such as palm leaf manuscripts [182], ensuring clearer visual data for researchers and curators. Machine learning techniques were also applied to address preservation challenges for artworks, focusing on detecting and mitigating damages caused by environmental factors or human activities [136]. Furthermore, AI algorithms combining techniques like Histogram of Oriented Gradients (HOG) and CNNs, were developed for classifying complex calligraphy styles, showcasing the advanced application of deep learning in managing intricate visual features in cultural heritage [91,170]. These interdisciplinary approaches, combining real-time data acquisition through IoT devices with automated recognition and restoration tools powered by AI, are set to transform heritage preservation and analysis. By integrating AI and IoT, curators and researchers can monitor environmental changes, track the structural integrity of sites, and perform restorations with unprecedented accuracy, ensuring the long-term sustainability of cultural heritage assets.

Overall, the development of image recognition technologies in cultural heritage has evolved through several interconnected areas, each expanding and refining the capabilities of the field. In digital documentation and retrieval, advancements in deep learning and AI have enabled more precise classification, recognition, and restoration of cultural heritage artifacts, enhancing both educational and preservation efforts. Archaeological analysis has seen significant progress with the integration of deep learning models and machine learning techniques, allowing for the automated detection and analysis of both surface and subsurface features, which supports more efficient archaeological surveys and prospection. For heritage object recognition, there has been a marked shift toward employing AI and deep learning models to handle complex visual features and to automate the recognition of diverse cultural symbols and styles. Meanwhile, the development of interactive applications has introduced new possibilities for engaging users with cultural heritage through IoT-based environments and tailored experiences, enriching the way heritage is understood and appreciated. Each of these areas has benefitted from the rapid advancements in deep learning, algorithm optimization, and interdisciplinary collaboration, which collectively drive the field toward more comprehensive, accurate, and accessible heritage preservation and analysis.

### 4.4. Future Research Directions

Future research directions in cultural heritage image recognition will likely focus on deeper integration of multidisciplinary approaches, incorporating the topics with the development potential from the third sub-period: “ecosystem services”, “patterns”, “biodiversity”, “policy”, “empowerment”, “science”, “algorithm”, and “energy”. The potential for these keywords indicates a shift toward combining AI and machine learning with ecological monitoring, sustainable energy management, and policy-making.

Based on the analysis of citation bursts and thematic evolution in cultural heritage image recognition research, several promising directions can be predicted for future studies. First, the continued rise in citations for “cultural heritage” and “world heritage” underscores a sustained focus on heritage management and digital preservation, with a particular emphasis on applying AI and deep learning techniques to handle complex visual and historical features [21,140,169]. With the recent rise in citations for “deep learning” and “knowledge”, there is an emerging interest in applying state-of-the-art machine learning models, such as Deep Residual Networks, to solve specific challenges like heritage object recognition and historical document analysis [106,177,179].

Second, a growing interdisciplinary trend is evident, where environmental science, policy-making, and energy management intersect with cultural heritage preservation. The increasing citation bursts for “ecosystem services” and “indigenous people” indicate a growing focus on using AI to support ecosystem services for heritage sites and to explore heritage’s relationship with indigenous knowledge systems [75,104,105]. The application of AI in these contexts could lead to more sustainable and inclusive heritage preservation approaches.

Additionally, research is expanding into areas like heritage tourism and urban planning, as image recognition technologies enhance visitor experiences and improve site management, as indicated by the recent burst in citations for “tourism” [180]. Further development in non-invasive techniques, such as material classification and the detection of environmental and human-induced damage, will continue to support the preservation of cultural artifacts [136,182].

As the field moves toward more specialized research, optimizing AI algorithms and computational methods will remain a key focus. This includes exploring new AI frameworks to improve recognition accuracy, data analysis, and predictive modeling in various heritage contexts. Researchers will likely combine different AI models to achieve higher precision in handling intricate visual features, as demonstrated by recent work on calligraphy and manuscript recognition [91,181].

Researchers are expected to explore how AI can be further integrated with ecological monitoring and energy management at heritage sites, while also considering the role of social policies and public participation in heritage preservation [183]. The combination of AI, deep learning, and big data technologies presents opportunities for real-time heritage monitoring, sustainable site management, and the development of user-friendly tools for heritage analysis. Public involvement will also be essential for creating more inclusive and effective heritage management approaches [184,185]. This study’s interdisciplinary approach to mapping the intersection of AI technologies, environmental science, and cultural heritage has highlighted previously unexplored areas. Bibliometric techniques have revealed important connections between emerging fields and offer a foundation for future research in this interdisciplinary space. By addressing this research gap, the study advances our understanding of cultural heritage preservation but also provides a roadmap for future innovations that span multiple disciplines.

In summary, future research will likely expand AI’s application in interdisciplinary areas, refining algorithms for greater precision and integrating ecological and social dimensions into heritage management. These advancements will address complex challenges related to heritage preservation and sustainability, promoting a more holistic and inclusive approach to the field. However, several limitations still need to be addressed. One major challenge is the computational cost of deep learning models, which requires substantial resources for training and deployment, limiting access for smaller institutions lacking infrastructure. Additionally, the need for large labeled datasets in cultural heritage remains an issue due to the incomplete or fragmented nature of heritage data. Future research should focus on reducing these costs, developing more efficient algorithms, and leveraging cloud-based infrastructures to make advanced tools more accessible.

Additionally, the over-reliance on AI in decision-making for heritage conservation raises ethical concerns. While AI models assist in analyzing cultural heritage, the human aspect—particularly cultural and contextual knowledge—must not be neglected. Future research should aim to develop hybrid models that blend automated processes with expert human input, ensuring that technological solutions remain sensitive to cultural and ethical considerations. Finally, making AI and IoT technologies more accessible, especially in regions with fewer resources, should be a priority. Developing scalable solutions that can be applied across diverse settings will help ensure these technologies have a global impact on heritage preservation. 

### 4.5. Research Limitations

There are several limitations to this study. First, the dataset was exclusively gathered from the Web of Science Core Collection, focusing primarily on English language publications. This limited scope may have excluded relevant research from other databases like Scopus, Google Scholar, or non-English sources, potentially narrowing the diversity of perspectives and research coverage. Future studies would benefit from integrating multiple databases to obtain a broader more comprehensive view of the research landscape.

Secondly, while the study applied bibliometric techniques using tools such as CiteSpace, Bibliometrix, and VOSviewer to analyze trends and collaborations, these methods primarily focused on abstracts, keywords, and titles. A more in-depth content analysis of full-text articles could reveal additional insights into nuanced methodologies and case studies. Another limitation stems from the exclusive use of the LLR algorithm for cluster analysis. Employing multiple algorithms may improve accuracy and provide a more detailed understanding of the evolving research themes.

Finally, the study’s focus on key clusters and citation bursts leaves other emerging or less prominent themes underexplored. While this allows for a more concise presentation, it may overlook smaller, yet significant, areas of inquiry that could shape future research directions. Future analyses could delve deeper into these peripheral clusters to uncover new research frontiers.

## 5. Conclusions

Bymploying bibliometric techniques, along with qualitative and quantitative analysis, this study explores the key developments and potential future research directions in cultural heritage image recognition from 1995 to 2024. The findings reveal a gradual evolution in the field, shifting from theoretical and macro-level research to more specific digital applications and technological integration, particularly through the use of advanced AI and deep learning techniques. This trend not only reflects the growing focus on digital preservation but also highlights the interdisciplinary nature of the field, combining insights from environmental science, policy-making, and algorithm optimization. Based on the findings, several key conclusions can be drawn, as follows:

- Evolution of Research Stages: Cultural heritage image recognition research can be divided into four distinct phases. The initial development phase (1995–2005) laid the groundwork for applying image recognition technology to cultural heritage analysis. The second phase (2006–2014) expanded these efforts, integrating feature extraction and classification techniques. The third phase (2015–2019) saw the maturation of machine learning applications, while the current phase (2020–2024) has been characterized by the rapid adoption of AI, deep learning, and interdisciplinary approaches;
- Key Research Themes: The primary themes in this field include cultural heritage management, deep learning-based recognition, and the application of AI in archaeological analysis and historical document preservation. During the third sub-period (2020–2024), research has focused on optimizing digital documentation, improving object recognition accuracy, and integrating IoT-based interactive applications for enhanced user engagement. Additionally, recent research shows increased interest in integrating AI models with ecological and social dimensions, exploring areas such as ecosystem services, policy-making, and sustainable management;
- Interdisciplinary Integration: The interdisciplinary nature of cultural heritage image recognition has become increasingly evident. Researchers are now incorporating insights from environmental science, policy, and social dimensions to address complex heritage preservation challenges. Emerging themes such as “ecosystem services”, “biodiversity”, “patterns”, “policy”, “empowerment”, “algorithm”, and “energy” reflect this shift toward a broader more integrated approach to heritage conservation.

## Figures and Tables

**Table 1:** Publications Co-citation Network Analysis.

CA	COL	SZ	RD	CP
Cluster1	red	11	Computer Science and Computer Vision	Proceedings of the IEEE Conference on Computer Vision and Pattern Recognition (ISSN: 1063-6919)
Cluster2	green	10	Tourism Management, Cultural Heritage Research, and Sustainability	International Journal of Heritage Studies (IJHS) (ISSN: 1352-7258)
Cluster3	blue	6	Interdisciplinary and Scientific Research	Science (ISSN: 0036-8075)
Cluster4	yellow	5	Cultural Heritage and Analytical Chemistry	Journal of Cultural Heritage (JCH) (ISSN: 1296-2074)
Cluster5	purple	3	Anthropology, Ethnobotany, and Food Chemistry	Journal of Ethnobiology and Ethnomedicine (ISSN: 1746-4269)

Data come from WoSCC and analysis results of VOSviewer. CA: Cluster Analysis; COL: Color; SZ: Size; RD: Research Direction; CP: Corresponding Publication.

**Table 2:** The dual-map overlay for related publications analysis.

PN	COL	LM	RM
Path 1	Red	Mathematics, Systems, Mathematical	Systems, Computing, Computer
Path 2	Dark Blue	Ecology, Earth, Marine	Plant, Ecology, Zoology
Path 3	Blue	Economics, Economic, Political	Economics, Economic, Political
Path 4	Cyan	Psychology, Education, Health	Psychology, Education, Social

Data come from WoSCC and analysis results of Citespace. PN: Path Number; COL: Color; LM: Left Map; RM: Right Map.

**Table 3:** Keywords with the strongest links.

R	C	K	O	TLS	AP
1	2	cultural-heritage	73	72	2020
2	2	heritage	51	77	2018
3	2	recognition	41	68	2018
4	1	management	27	69	2019
5	3	tourism	22	42	2020
6	1	conservation	21	33	2020
7	2	politics	20	33	2018
8	3	culture	16	20	2019
9	1	unesco	16	35	2020
10	5	world heritage	16	27	2019

Data come from WoSCC. R: Ranks; C: Clusters; K: Keywords; O: Occurrences; TLS: Total Link Strength; AP: Years on average since publication.

**Table 4:** Keyword cluster: Top 5 Clusters.

ID	CL	SZ	SIL	M (Y)	TT (LLR, P-Level)
0	Cultural Heritage	52	0.973	2019	cultural heritage (21.12, 1.0E−4); deep learning (18.14, 1.0E−4); machine learning (15.52, 1.0E−4); computer vision (12.91, 0.001); digital humanities (10.31, 0.005)
1	Climate Change	42	0.92	2016	climate change (12.09, 0.001); intangible heritage (4.5, 0.05); historic cities (4.01, 0.05); globally important agriculture heritage system (4.01, 0.05); geoconservation (4.01, 0.05)
2	Pollution	40	0.94	2011	pollution (4.25, 0.05); hudewald (4.25, 0.05); rights to the city (4.25, 0.05); impact (4.25, 0.05); biobanks (4.25, 0.05)
3	Politics	38	0.956	2014	politics (9.86, 0.005); contemporary heritage protection (4.91, 0.05); palestinian citizens (4.91, 0.05); australia-oceania region (4.91, 0.05); womens work (4.91, 0.05)
4	Urban Tourism	34	0.949	2014	urban tourism (11.07, 0.001); life cycle sustainability assessment (5.51, 0.05); urban sociology (5.51, 0.05); deaf (5.51, 0.05); smooth space (5.51, 0.05)

Data come from WoSCC and analysis results of Citespace. ID: Cluster ID; CL: Cluster Label; SZ: Size; SIL: Silhouette; M (Y): Mean (Year); TT: Top Terms; LLR: log-likelihood ratio.

**Table 5:** The top 10 most frequently occurring keywords from 2020 to the present.

Freq	B	BB	BE	C	Label	Cluster ID
73	6.11	2022	2024	0.2	cultural heritage	0
24	1.48	2023	2024	0.22	recognition	5
13	2.71	2020	2024	0.04	world heritage	7
13	3.09	2022	2024	0.02	deep learning	0
12	0.9	2023	2024	0.01	intangible cultural heritage	1
10	2.18	2021	2024	0.01	indigenous people	2
7	0.8	2023	2024	0.05	framework	4
7	1.57	2022	2024	0.11	archaeology	3
6	0.89	2023	2024	0.03	city	4
6	1.58	2023	2024	0.04	science	10

Data come from WoSCC and analysis results of Citespace. Freq: Frequency; B: Burst; BB: Burst Begin; BE: Burst End; C: Centrality; ID: Cluster ID.

## Data Availability

Data are contained within the article.
